# SERVICE LEARNING AS A MEANS TO ENHANCE CONNECTIONS TO OLDER ADULTS

**DOI:** 10.1093/geroni/igz038.1969

**Published:** 2019-11-08

**Authors:** Marjorie A Getz

**Affiliations:** Methodist College, Peoria, Illinois, United States

## Abstract

Aging is a distinct part of the life cycle. College students enrolled in courses in gerontology often have difficulty relating to aging, that part of life not yet experienced. They may not fully appreciate that adults become more unique, not more similar, as they age. We describe courses in an undergraduate gerontology certificate program that incorporate experiential learning activities with older adults across a hierarchical sequence of courses. These courses feature service learning opportunities focused on increased understanding of course content, broader appreciation of the discipline and improved sense of civic responsibility. Much like the course content of the curriculum, the incorporated experiential learning opportunities for each course level fit a hierarchy leading to student competence and skills development needed for success in the final independent practicum. For the described courses, students provided community service, experienced direct contact with older adults and used reflective practices to integrate course content into service learning activities. We report on qualitative data obtained from students enrolled in the foundational course, Biophysical Aspects of Aging and the third level course, Aging and Mental Health. Content analyses of reflective essays identified five themes: (a) insights about the realities of aging in America (b) perceptions concerning personal negative stereotypes about older adults; (c) feelings of accomplishment/awareness of new skills in providing community services; (d) understandings related to the importance/value of community service; and (e) successes in integrating the course work on aging into service-learning experiences. Other experiential learning activities incorporated into this gerontology certificate program are highlighted.

